# *Bombyx mori p38 MAPK* Regulates Cell Cycle Progression and Silk Gland Endoreplication via Interaction with Cell Cycle Genes

**DOI:** 10.3390/insects17101006

**Published:** 2026-09-30

**Authors:** Lanxing Wang, Yanbi Long, Siyi Wei, Keqin Ding, Mingli Zhang, Yan Zhu, Zhanqi Dong, Cheng Lu, Peng Chen, Minhui Pan

**Affiliations:** 1 State Key Laboratory of Resource Insects, Southwest University, Chongqing 400715, China; 2 Key Laboratory of Sericultural Biology and Genetic Breeding, Ministry of Agriculture and Rural Affairs, Southwest University, Chongqing 400715, China

**Keywords:** *Bombyx mori*, cell cycle, cyclins, endoreplication, *p38 MAPK* gene

## Abstract

The cell cycle is a fundamental biological process indispensable for life, and its orderly progression is governed by a complex signaling network. Although the p38 MAPK pathway plays important roles in regulating cell proliferation across metazoans, its mechanistic role in insect cell cycle progression remains poorly characterized, particularly in species with endoreplication systems. Here, we investigated the function and mechanism of *Bombyx mori p38 MAPK* (*Bmp38 MAPK*) in cell cycle regulation using the silkworm silk gland as a specialized endoreplication model. We found that the p38 MAPK is evolutionarily conserved from insects to mammals and exhibits both cytoplasmic and nuclear localization, with its expression peaking during the G1 phase. Overexpression of *Bmp38 MAPK* induced cell cycle arrest at both G1/S and G2/M phases and suppressed cell proliferation, whereas *Bmp38 MAPK* knockdown alleviated G1/S arrest and enhanced proliferation. Mechanistically, *Bmp38 MAPK* increased *BmCyclin B* expression while reducing the expression of *BmCyclin E*, *BmCDK2* and *BmCDK1*. Protein interaction assays further confirmed interactions between Bmp38 MAPK and BmCDK2, BmCyclin E and BmCyclin B. Notably, *Bmp38 MAPK* significantly inhibited DNA synthesis in silk gland cells, suggesting that *Bmp38 MAPK* contributes to the regulation of endoreplication. These findings reveal a possible role for p38 MAPK in insect cell cycle control and provide potential mechanistic insights into the regulation of endoreplication in the silkworm silk gland.

## Simple Summary

The p38 MAPK pathway plays an important role in cell proliferation and survival, but its function in insect cell cycle regulation remains poorly understood. Here, we investigated the role and mechanism of *Bombyx mori p38 MAPK* in the cell cycle progression and endoreplication. Overexpression of *Bmp38 MAPK* induced G1/S and G2/M cell cycle arrest and suppressed cell proliferation, whereas its knockdown promoted proliferation. Mechanistically, *Bmp38 MAPK* increased *BmCyclin B* expression while reducing *BmCyclin E*, *BmCDK2* and *BmCDK1* expression. Protein interaction assays further demonstrated its association with BmCDK2, BmCyclin E, and BmCyclin B. Notably, *Bmp38 MAPK* inhibited DNA replication in silk gland cells, suggesting a role in regulating endoreplication. These findings reveal the possible mechanism of p38 MAPK in insect cell cycle regulation and provide insights into the coordination of endoreplication.

## 1. Introduction

Cell proliferation is a fundamental process governing growth and development. The cell cycle, being the cornerstone of life processes, plays a pivotal role in regulating cell proliferation and division. A typical mitotic cell cycle is divided into four phases: G1 phase, S phase, G2 phase and M phase [1]. Proper regulation of multiple factors, including classical signaling pathways like MAPK, Wnt/β-catenin, Notch, and Hippo, is crucial for ensuring smooth and orderly progression of the cell cycle [2,3,4,5]. The MAPK signaling pathway is among the most highly conserved signaling pathways and plays a key role in the regulation of cell proliferation, encompassing four distinct signaling pathways, namely p38, ERK1/2, JNK and ERK5 [6,7,8].

As an important member of the MAPK family, p38 MAPK is involved in various cellular processes. In addition to its established roles in apoptosis and cellular stress responses, p38 MAPK also plays an important role in cell cycle regulation [9,10,11]. In mammals, the p38 MAPK family consists of four major isoforms: p38α, p38β, p38γ and p38δ [12,13]. Both p38α and p38γ have been reported to regulate cell cycle progression through the retinoblastoma protein (RB), although their effects occur at different cell cycle stages. p38α induces G1 phase arrest, whereas p38γ promotes G2/M phase progression. Additionally, p38 can cause the degradation of cyclin D by phosphorylation, thereby mediating G1 phase arrest [14,15,16,17]. During the G2 phase, activated p38 MAPK can inhibit CDC25 activity, resulting in sustained CDK1 inhibition and G2 phase arrest [10]. The *Drosophila* genome encodes three p38 homologs, p38a, p38b and p38c [18,19]. However, the role of p38 in the insect cell cycle remains poorly understood, and the underlying mechanisms require further investigation.

The silkworm, *Bombyx mori*, is an important economic insect of the Lepidoptera family. The silk gland is a unique organ in silkworms, responsible for the synthesis and secretion of silk protein [20,21,22]. During growth and development, silk gland cells follow a specialized cell cycle program. At embryonic stage 24, silk gland cells undergo mitosis, begin transitioning from mitosis to endoreplication at stage 25, and undergo endoreplication from stage 26 onward. Endoreplication is a specialized cell cycle program in which DNA is repeatedly replicated without subsequent cell division, resulting in progressive increases in cellular ploidy [23,24,25]. This process is essential for the efficient production of silk proteins by enabling the silk gland cells to support sustained transcriptional and biosynthetic activity. Thus, investigating the cell cycle regulation of silkworm cells and silk glands holds immense significance. In this study, we aimed to elucidate the function and mechanism of the *p38 MAPK* gene in regulating the cell cycle and endoreplication in the silkworm. We confirmed the suppressed function of the *Bmp38 MAPK* gene in DNA replication of silk gland tissue in vitro by interacting with cyclins. Our results provide evidence for the role of *Bmp38 MAPK* in the cell cycle of silkworm and provide a potential molecular basis for unraveling the mechanism of endoreplication of silk gland cells.

## 2. Materials and Methods

### 2.1. Silkworm and Cell Line

The Dazao silkworm larvae were fed with fresh mulberry leaves and housed under a 12 h light/12 h dark photoperiod at 25 °C. BmE-SWU1 cells [26] were cultured at 27 °C in Grace insect medium (Gibco, Grand Island, NY, USA) supplemented with 10% fetal bovine serum (FBS, BIOAGRIO, Mountain View, CA, USA).

### 2.2. Phylogenetic Analysis and Conserved Motifs

The National Centre for Biotechnology Information (NCBI) website was used to obtain 37 p38 MAPK protein sequences from different species. A phylogenetic tree of p38 MAPK was constructed using MEGA6.0 with the neighbor-joining method. Multiple sequence alignments were performed to identify the conserved motifs of the p38b in insects and vertebrates. The conserved motifs of the proteins were analyzed online using MEME software version 5.5.1. The motif structures and the phylogenetic tree were drawn by TBtools (version 1.108) [27].

### 2.3. Plasmid Construction

The *p38 MAPK* gene was cloned into the pIZ/V5-His-EGFP vector to obtain an overexpression plasmid. RNA interference sequences were designed by the Invitrogen BLOCK-iT^TM^ RNAi Designer website. Three small interfering RNA sequences with discontinuous positions were inserted into the endogenous microRNA skeleton of a silkworm and cloned into the pIZ/V5-His-EGFP to construct the interference vector. The primer sequences used in this study are listed in Table S1.

### 2.4. In Vitro Culture of Silk Glands and Transient Transfection

Silk gland tissues were dissected from silkworms at the second day of the fourth instar (4L2D) and washed twice with PBS to remove residual hemolymph and debris. The isolated tissues were subsequently transferred to 12-well plates containing 1 mL of Grace insect medium (Gibco) supplemented with 10% FBS (BIOAGRIO) per well and maintained at 27 °C. For transient transfection, the plasmid was mixed thoroughly with the insect transfection reagent (Mirus, Madison, WI, USA) at a DNA-to-reagent ratio of 1:2, with 1 μg of plasmid used per well. The mixture was added to the cultured silk gland tissues. After 48 h of incubation, the tissues were collected for subsequent analyses.

### 2.5. Quantitative Real-Time Polymerase Chain Reaction (qRT-PCR)

RNA was extracted by the Total RNA kit (Omega Bio-Tek, Norcross, GA, USA) and cDNA was synthesized with the RT Reagent Kit (Takara, Dalian, China). qRT-PCR was conducted by SYBR qPCR Supermix Plus (Novo-protein, Shanghai, China) using the real-time PCR system (Bio-Rad, Hercules, CA, USA). The *eukaryotic translation initiation factor 4A* gene (sw22934) was used as the internal control. All qRT-PCR data were analyzed using the 2^−ΔΔCt^ method. Relevant primer sequences are listed in Table S1.

### 2.6. Cell Counting Kit-8 Assay (CCK-8)

A CCK-8 reagent was used to detect the proliferation activity of the cells at 0 h, 24 h and 48 h after plasmid transfection. The cell suspension was divided into 96-well culture plates at 100 μL per well in triplicate, and an additional 10 μL of CCK-8 solution (Yeasen Biotechnology, Shanghai, China) was added to each well. The cell culture plate was placed in an incubator at 37 °C for 2 h. OD values were measured at a wavelength of 540 nm.

### 2.7. Flow Cytometry Analysis

After transfection, cells were washed with PBS and then fixed with 70% ethanol at −20 °C overnight. The ethanol was discarded by low-speed centrifugation. RNase A and propidium iodide (PI) were added following the cell cycle and apoptosis analysis kit instructions (Beyotime, Shanghai, China). Cell samples were subjected to cell cycle analysis using flow cytometry (CYTOFLEX, Beckman Coulter, Brea, CA, USA).

### 2.8. Cell Synchronization

Cell cycle synchronization of BmE-SWU1 cells was achieved using phase-specific chemical treatments. G1/S phase enrichment was established by aphidicolin (Sigma, St. Louis, MO, USA) at 5 mg/mL for 24 h [28]. To obtain an S phase-enriched population, cells were incubated with 1 mM hydroxyurea (Sigma, St. Louis, MO, USA) for 16 h and subsequently transferred to hydroxyurea-free medium for an additional 7 h [29]. G2/M phase synchronization was performed by treating the cells with nocodazole (Sigma, St. Louis, MO, USA) at 10 mg/mL for 24 h [30].

### 2.9. 5-Ethynyl-2′-Deoxyuridine (EdU) Assay and Immunofluorescence Stain

The BmE-SWU1 cells and silk glands were cultured for 48 h after transfection with different plasmids and then for 2 h with the EdU reagent according to the BeyoClick™ EdU cell proliferation kit (Beyotime). Cells and silk glands were treated with 4% paraformaldehyde (Beyotime) and 1% TritonX-100 (Beyotime) for 15 min at room temperature and then incubated with primary antibodies, anti-EGFP (1:200; Beyotime), anti-FLAG (1:200; Abmart, Shanghai, China), and secondary antibodies, Alexa Fluor 555 (1:500; Thermo Fisher Scientific, Waltham, MA, USA) for 1 h at 37 °C. Nuclei were stained with Hoechst (1:500; Thermo Fisher Scientific). Images were taken using a confocal microscope FV3000 (Olympus, Tokyo, Japan), and the number of positive cells was counted by Photoshop software (version 2018).

### 2.10. Co-Immunoprecipitation (CO-IP) and Western Blotting Assay

Cell samples were collected 48 h after transfection of 3 μg of the corresponding plasmid in BmE-SWU1 cells. The cells were lysed by the addition of cell lysis buffer (Beyotime) and protease inhibitor (PMSF) on ice for 30 min. Subsequently, the lysates were centrifuged at 13,000× *g* for 10 min for clarification, and the supernatant was harvested. Flag antibody (1:100; Abmart) and immunoglobulin G (IgG) antibody (1:100; Abmart) were added and incubated overnight at 4 °C on a rotating shaker. Protein A/G magnetic beads were added to facilitate antibody binding, and the eluate was collected. The collected samples were mixed with 5 × SDS loading buffer and separated by 12% SDS-PAGE after 10 min in a water bath at 100 °C. Proteins were subsequently transferred to hydrophilic polyvinylidene fluoride (PVDF) membranes, incubated with primary antibodies, anti-Flag (1:2000; Abmart), anti-Myc (1:2000; Abmart), anti-HA (1:2000; Bioworld, St. Louis Park, Minnesota, USA), anti-α-Tubulin (1:2000; Beyotime), and secondary antibodies (1:5000; Beyotime). The band was visualized using Western ECL luminescent solution (Yeasen Biotechnology).

### 2.11. Yeast Two-Hybrid (Y2H) Assay

The coding DNA sequence (CDS) of the bait protein was cloned into the pGBKT7-Bait vector, while the CDS of the prey protein was inserted into the pGADT7-Prey vector, the corresponding primers listed in Table S1. The two recombinant plasmids were co-transformed into competent AH109 yeast cells according to the kit (Coolaber, Beijing, China) and spread evenly on SD/-Trp/-Leu solid medium for the selection of co-transformants. Positive co-transformants were diluted 100-fold and streaked onto SD/-Trp/-Leu/-His and SD/-Trp/-Leu/-His/-Ade solid media to verify the interaction between the bait and prey proteins.

### 2.12. Statistical Analysis and Image Processing

All experiments were performed with three independent biological replicates. Statistical analysis was performed using GraphPad Prism software (Version, 8.0). For comparisons involving the same samples measured under two different conditions, a paired Student’s *t*-test was used to assess statistical significance. Data are presented as mean ± SD. *** *p* < 0.001; ** *p* < 0.01; * *p* < 0.05; ns, not significant. All figures were assembled and arranged using Adobe Illustrator (Version, 2026).

## 3. Results

### 3.1. Identification and Expression Pattern Analysis of the Bmp38 MAPK Gene

**Figure 1:**
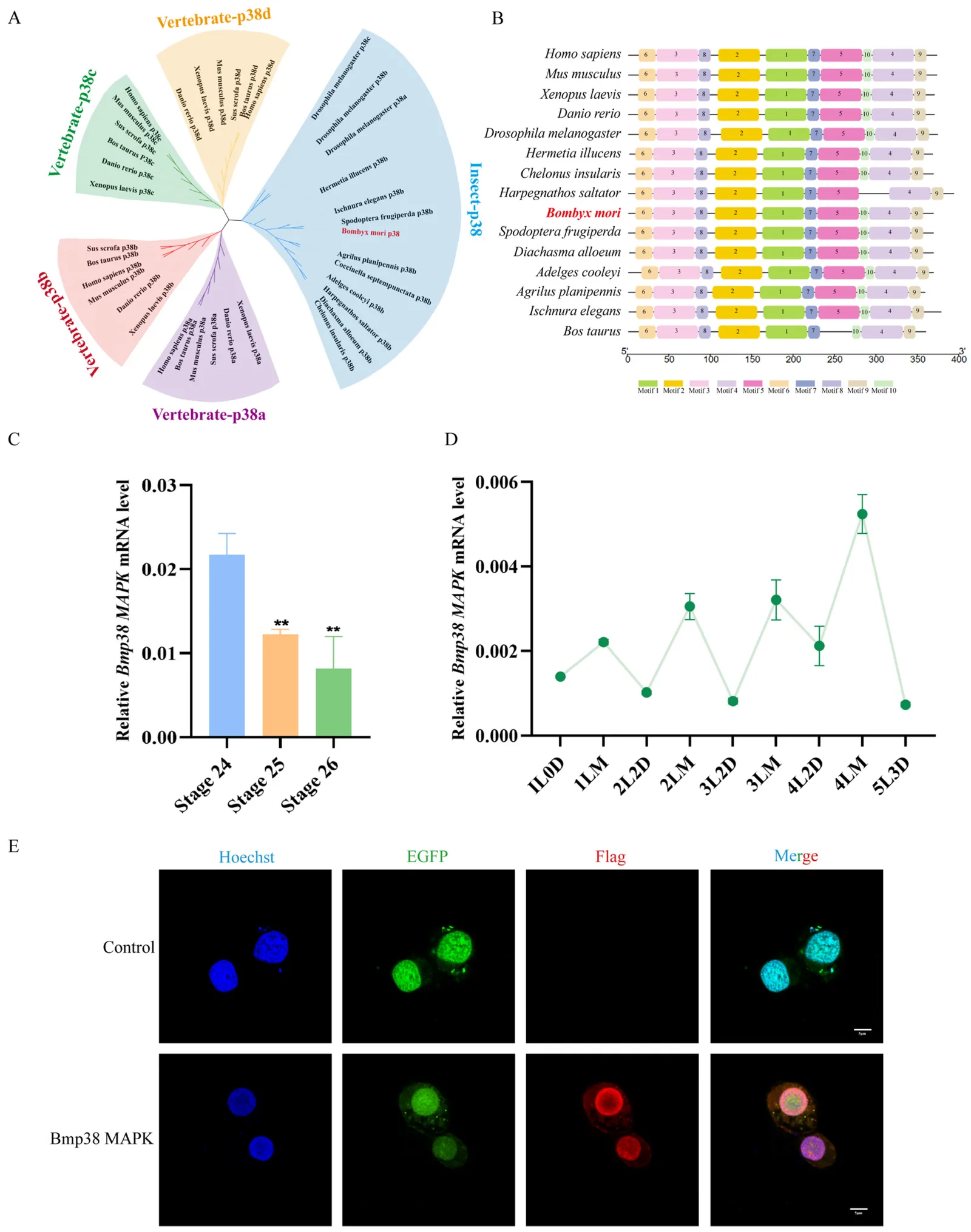
Phylogenetic analysis and expression pattern analysis of the *Bmp38 MAPK* gene. (**A**) The phylogenetic tree was constructed using p38 MAPK protein sequences by MEGA 6.0 software, using the neighbor-joining method. The analysis was carried out on 1000 bootstrapped datasets. The labels outside the phylogenetic tree indicate different isoforms and correspond to different colors. The red font represents the p38 MAPK protein of silkworm. (**B**) Distribution of conserved motifs of p38 MAPK protein in different species, with different colored boxes representing different conserved motifs. (**C**) The relative RNA expression levels of *Bmp38 MAPK* in the embryonic silk gland of the silkworm. (**D**) The relative RNA expression levels of *Bmp38 MAPK* in the silk gland during postembryonic developmental stages of the silkworm. (**E**) Immunofluorescence analysis of Bmp38 MAPK protein expression in BmE-SWU1 cells; scale bar = 5 μm. ** *p* < 0.01, paired Student’s *t*-test.

We cloned the *Bombyx mori p38 MAPK* gene from a silkworm cDNA library and performed a phylogenetic analysis in different species. The result showed that four p38 MAPK isoforms were present in vertebrates, each forming an independent clade. The sole p38 MAPK protein identified in the silkworm was classified within the p38b subtype and clustered with p38 MAPK homologs from other insect species (Figure 1A). Furthermore, we identified 10 conserved motifs and an S-TKc domain within the p38b protein sequences of insects and vertebrates (Figure 1B and Figure S1A). These results suggest the evolutionary conservation of this protein domain across these diverse groups. We further analyzed the spatiotemporal expression profile of the *Bmp38 MAPK* gene. The results demonstrated that *Bmp38 MAPK* exhibited high expression during the molting period, and low expression during the feeding period. The continuous low expression in the silk gland at the 24th, 25th and 26th stages also suggested that the *Bmp38 MAPK* gene had a negative regulatory effect on the endoreplication (Figure 1C,D). Immunofluorescence results indicated that the Bmp38 MAPK protein was localized in both cytoplasm and nucleus, which was consistent with the prediction of the Cell-PLoc 2.0 website (Figure 1E and Figure S1B). This provides a spatial basis for the execution of its regulatory function.

### 3.2. Bmp38 MAPK Gene Negatively Regulated the Cell Cycle Process

**Figure 2:**
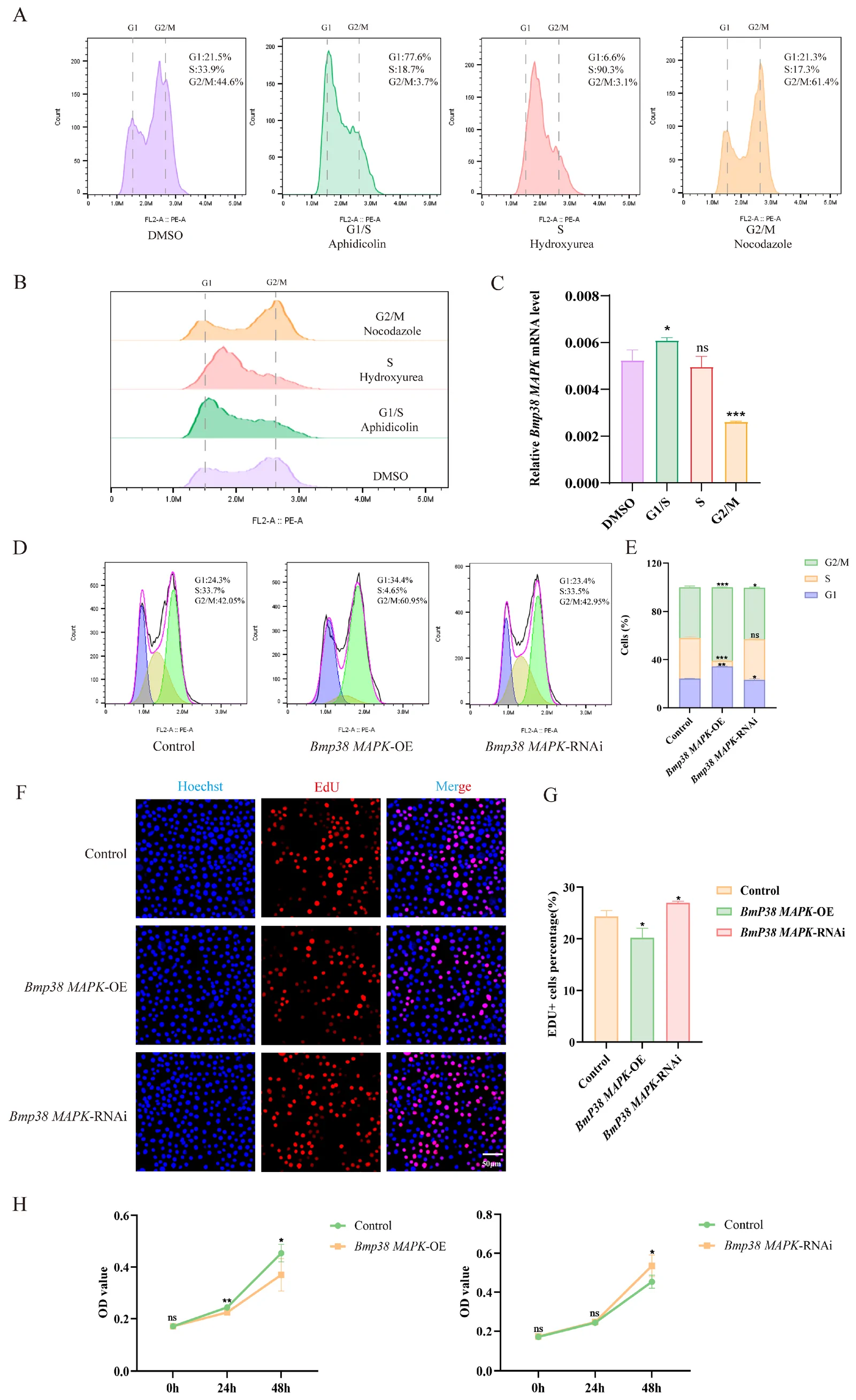
Functional analysis of the *Bmp38 MAPK* gene on cell cycle progression. (**A**,**B**) Synchronization reagents blocked the cell cycle of BmE-SWU1 cells: aphidicolin blocked the G1 phase of the cell cycle; hydroxyurea was used in the S phase of the cell cycle; nocodazole was used in the G2/M phase of the cell cycle. (**C**) Relative expression of *Bmp38 MAPK* at different cell cycle phases in BmE-SWU1 cells. (**D**) Cell cycle analysis of BmE-SWU1 cells by flow cytometry after overexpression and interference of the *Bmp38 MAPK* gene. (**E**) Statistical analysis of the percentage of cells in each phase of the cell cycle. (**F**) EdU labeling BmE-SWU1 cells. scale bar = 50 μm. (**G**) Statistical analysis of EdU positive cells. (**H**) CCK-8 reagent was used to detect the proliferation activity of BmE-SWU1 cells at 0, 24 and 48 h after plasmid transfection. *** *p* < 0.001; ** *p* < 0.01; * *p* < 0.05; ns, no significant difference, paired Student’s *t*-test.

To characterize the expression of the *Bmp38 MAPK* gene during the cell cycle phase, we examined the expression level of the *Bmp38 MAPK* gene after treatment with aphidicolin, hydroxyurea and nocodazole, which arrest the cell cycle in the G1/S, S and G2/M phases, respectively. Compared to the control group, the *Bmp38 MAPK* gene was most highly expressed in the G1 phase (Figure 2A–C), suggesting that it primarily functions in the G1 phase of the cell cycle. To identify the function of the *Bmp38 MAPK* gene on cell cycle progression, cells transfected with overexpression and interference plasmids for 48 h were collected for flow cytometry analysis. Overexpression of the *Bmp38 MAPK* gene resulted in a notable increase in G1 and G2/M phase cells and a concomitant reduction in S phase cells, in comparison with the controls. In contrast, interference of the *Bmp38 MAPK* gene decreased the proportion of cells in G1 and G2/M phases (Figure 2D,E and Figure S2A,B). To explore whether the *Bmp38 MAPK* gene affects DNA replication, we evaluated DNA replication efficiency by EdU labeling experiments. The EdU positive labeling rate was observably decreased after the overexpression of the *Bmp38 MAPK* gene, while the EdU positive labeling rate was observably increased after the interference of the *Bmp38 MAPK* gene (Figure 2F,G). Consistently, the CCK-8 assay showed that, compared with the control group, overexpression of the *Bmp38 MAPK* gene decreased cell proliferation activity, and interference of the *Bmp38 MAPK* gene increased cell proliferation activity (Figure 2H). These results demonstrate that the *Bmp38 MAPK* gene plays a negative regulating role in the cell cycle process.

### 3.3. Function of the Bmp38 MAPK Gene in DNA Synthesis in Silk Gland Cells

**Figure 3:**
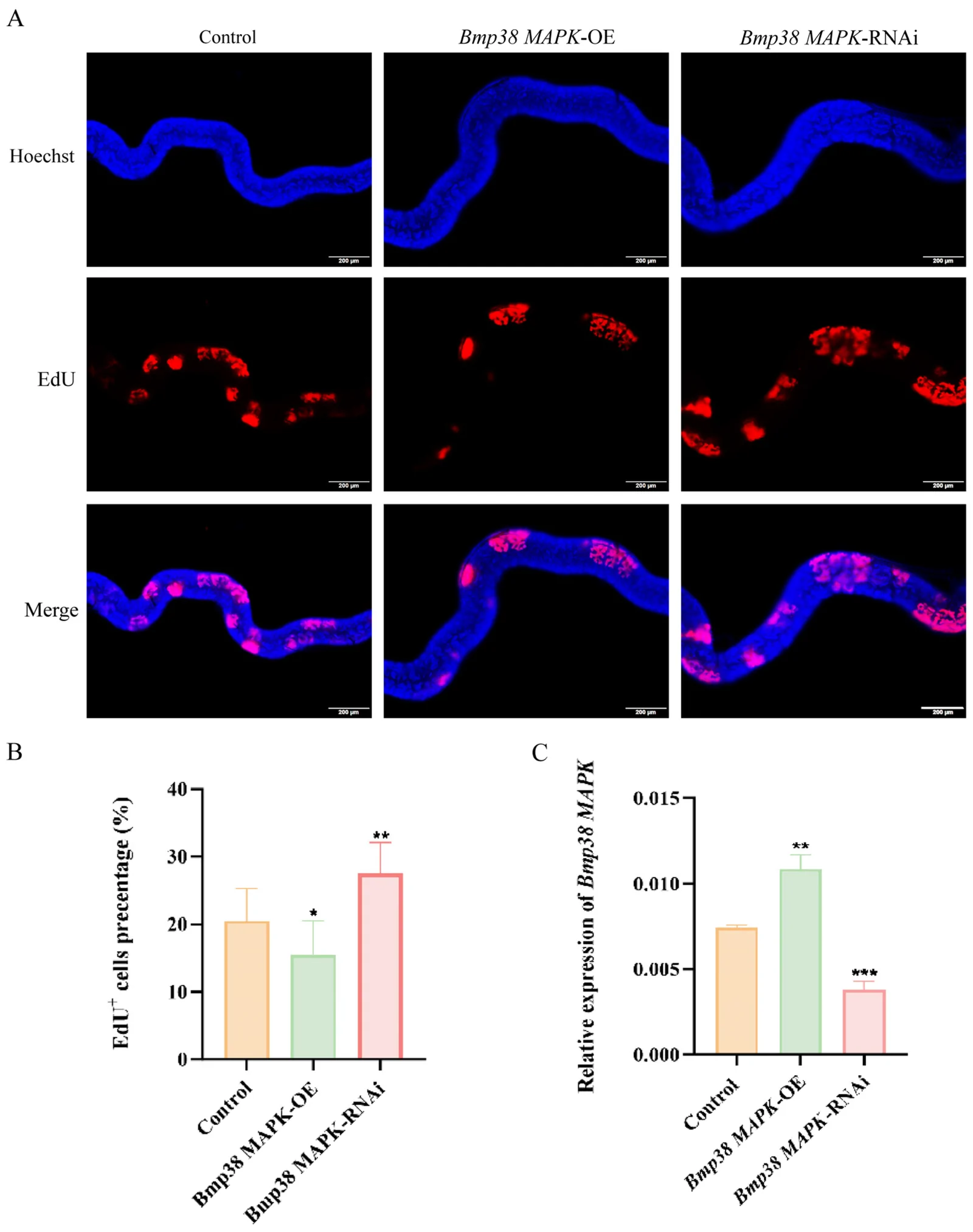
Analysis of the effect of the *Bmp38 MAPK* gene on DNA replication in silk glands. (**A**) Silk gland cells cultured in vitro were labeled with EdU. Red fluorescence represents EdU positive silk gland cells; scale bar = 200 μm. (**B**) Statistical analysis of EdU positive silk gland cells. Each value represents the mean ± SD (*n* = 3). (**C**) The relative RNA expression levels of *Bmp38 MAPK* in the silk gland after overexpression or interference of the *Bmp38 MAPK* gene. *** *p* < 0.001; ** *p* < 0.01; * *p* < 0.05, paired Student’s *t*-test.

To confirm the role of the *Bmp38 MAPK* gene in endoreplication in the silk gland tissue, we obtained the silk gland tissues from fourth-instar second-day silkworm larvae and cultured them in vitro. After transient transfection with the *Bmp38 MAPK* gene expression vector for 48 h, the efficiency of DNA synthesis was detected by EdU labeling assay. The results showed that interference with the *Bmp38 MAPK* gene expression greatly increased DNA replication in silk gland tissues whereas overexpression of the *Bmp38 MAPK gene* dramatically decreased DNA synthesis in those tissues (Figure 3A–C). These results indicate that the *Bmp38 MAPK* gene inhibits endoreplication in silk gland cells.

### 3.4. Effects of Bmp38 MAPK on Cell Cycle Genes

**Figure 4:**
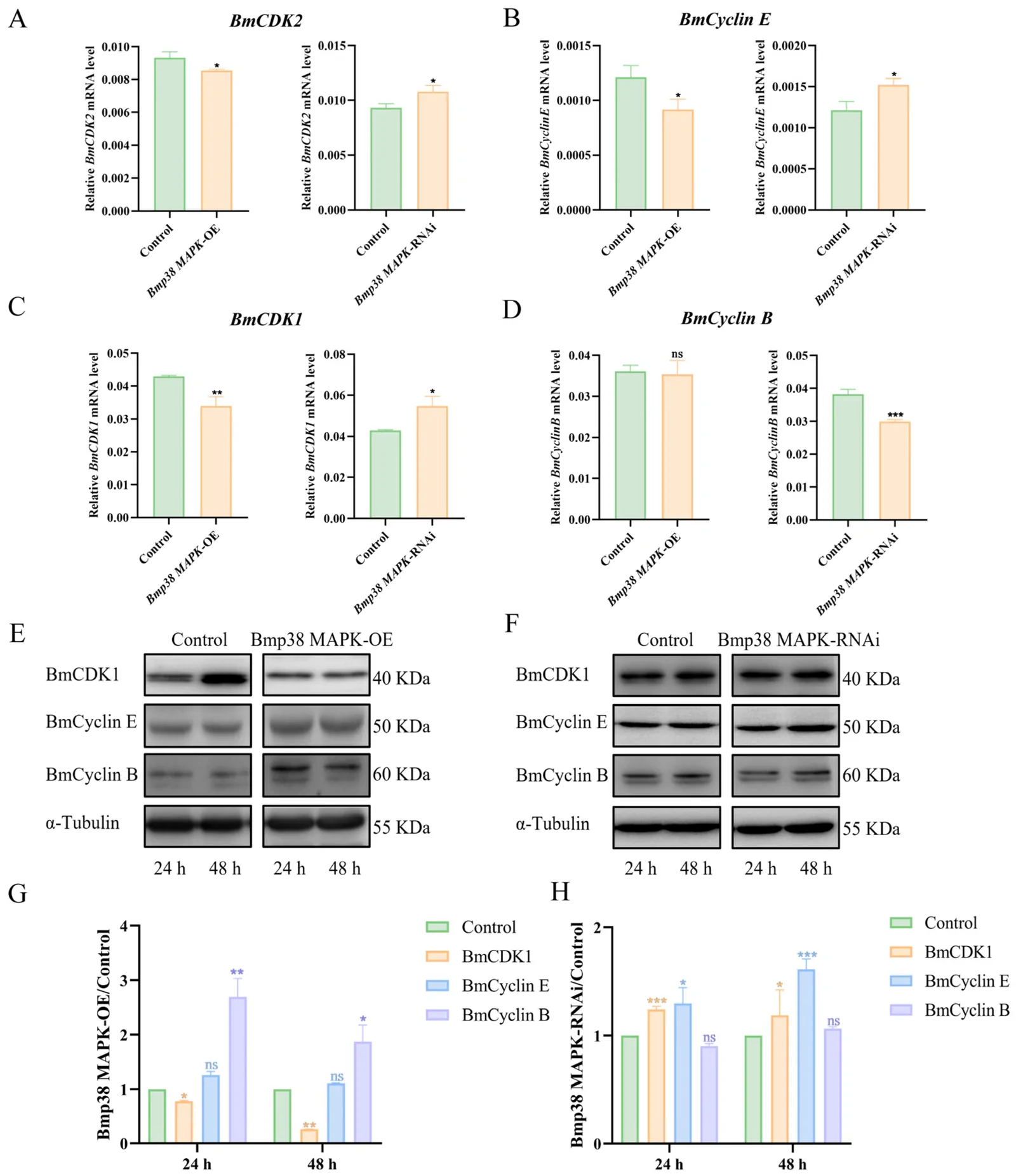
Effects of *Bmp38 MAPK* on cell cycle genes. (**A**–**D**) The relative RNA expression levels of *BmCDK2*, *BmCyclinE*, *BmCDK1 and BmCyclinB* in BmE-SWU1 cells after overexpression and interference of the *Bmp38 MAPK* gene. (**E**,**F**) The protein expression levels of BmCyclinE, BmCDK1 and BmCyclinB in BmE-SWU1 cells after overexpression and interference of the Bmp38 MAPK. (**G**,**H**) Statistical analysis of gray values. Each value represents the mean ± SD (*n* = 3). *** *p* < 0.001; ** *p* < 0.01; * *p* < 0.05, ns, no significant difference, paired Student’s *t*-test.

We sought to ascertain whether the genes *BmCyclin E*, *BmCDK2*, *BmCyclin B*, and *BmCDK1*, which are associated with the G1/S and G2/M checkpoints, are regulated by the *Bmp38 MAPK* gene. The data showed that overexpression of *Bmp38 MAPK* significantly downregulated the mRNA expression levels of *BmCyclin E*, *BmCDK2* and *BmCDK1*. Inversely, knockdown of *Bmp38 MAPK* markedly increased the mRNA expression levels of *BmCyclin E*, *BmCDK2* and *BmCDK1* (Figure 4A–D). The Western blotting results demonstrated that the overexpression of Bmp38 MAPK promoted the expression of the BmCyclin B protein and inhibited the expression of the BmCDK1 protein, whereas the interference of Bmp38 MAPK promoted the protein expression of BmCyclin E and BmCDK1 but did not affect the protein expression of BmCyclin B (Figure 4E–H). These results demonstrate that the *Bmp38 MAPK* gene regulates cell cycle progression by modulating the expression of cell cycle-related genes.

### 3.5. Identification of Proteins Interacting with Bmp38 MAPK

**Figure 5:**
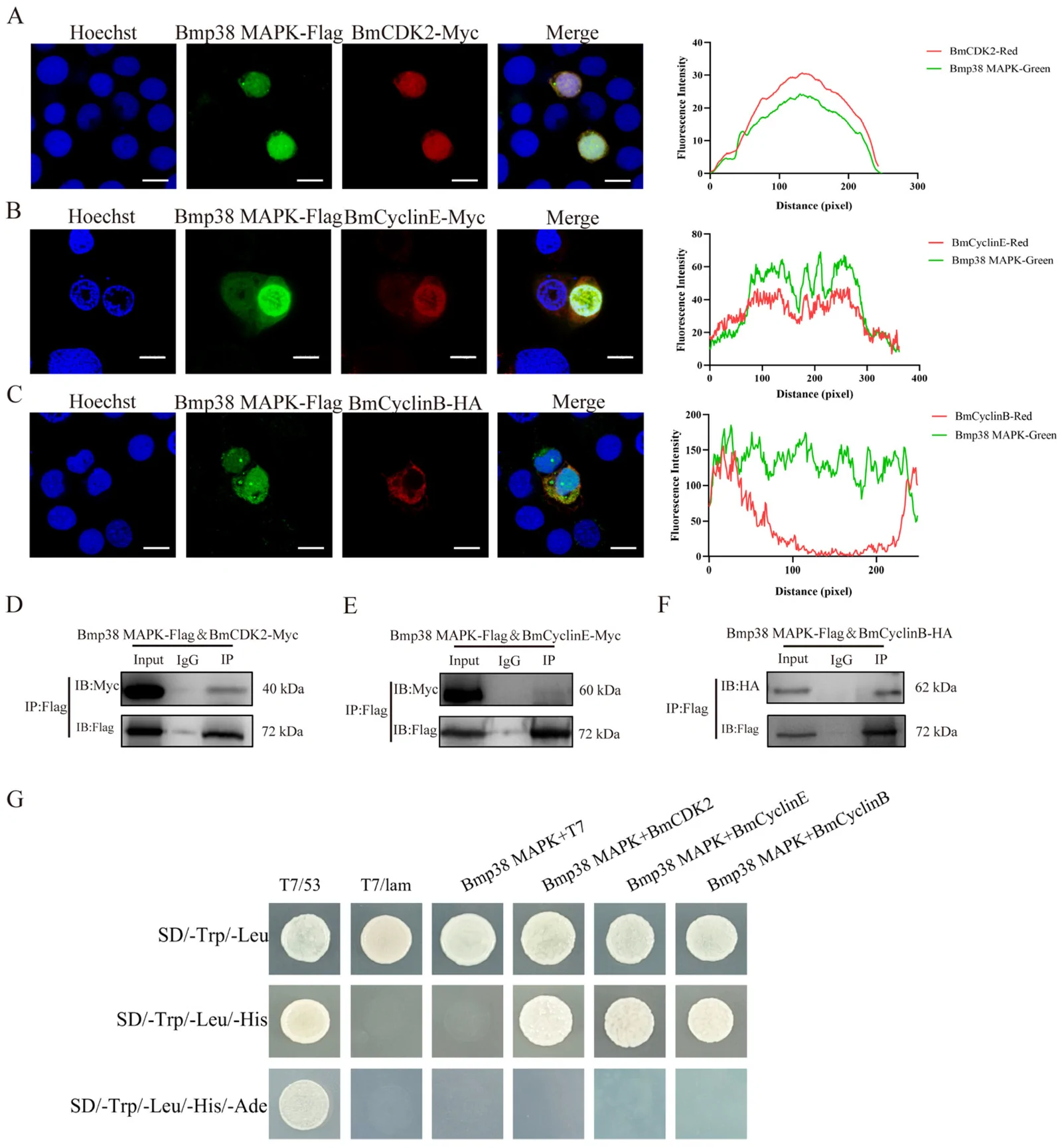
Identification of proteins interacting with Bmp38 MAPK. (**A**) Co-localization of Bmp38 MAPK and BmCDK2 in BmE-SWU1 cells. scale bar = 10 μm. Fluorescence co-localization analysis between Bmp38 MAPK and BmCDK2 with ImageJ software (version 1.53). (**B**) Co-localization of Bmp38 MAPK and BmCyclin E in BmE-SWU1 cells. scale bar = 10 μm. Fluorescence co-localization analysis between Bmp38 MAPK and BmCyclin E with ImageJ software (version 1.53). (**C**) Co-localization of Bmp38 MAPK and BmCyclin B in BmE-SWU1 cells. scale bar = 10 μm. (**D**) Fluorescence co-localization analysis between Bmp38 MAPK and BmCyclin B with ImageJ software (version 1.53). (**E**) Co-IP assay of Bmp38 MAPK and BmCDK2. (**F**) Co-IP assay of Bmp38 MAPK and BmCyclin E. (**G**) Co-IP assay of Bmp38 MAPK and BmCyclin B. (**G**) Yeast two-hybrid (Y2H) assay verified the direct interaction between Bmp38 MAPK and each of the three candidate proteins (BmCDK2, BmCyclin E, and BmCyclin B).

To elucidate the regulation mechanism of Bmp38 MAPK and cyclins, we constructed vectors of BmCyclin B, BmCyclin E, BmCDK2 and BmCDK1 that co-transfected with Bmp38 MAPK into cells for immunofluorescence, Co-IP and Y2H assay. The results showed that BmCDK2, BmCDK1 and BmCyclin E co-localized with Bmp38 MAPK in the cytoplasm and nucleus, and BmCyclin B co-localized with Bmp38 MAPK in the cytoplasm (Figure 5A–C and Figure S3A). The Co-IP and Y2H assay further confirmed that Bmp38 MAPK could interact with BmCDK2, BmCyclin B and BmCyclin E, but not with BmCDK1 (Figure 5D–G and Figure S3B,C). These results demonstrate that Bmp38 MAPK interacts with several cell cycle regulators and may contribute to the regulation of cell cycle progression through these interactions.

## 4. Discussion

The orderly operation of the cell cycle is the basis of organism proliferation and development, and more and more genes have been proved to be involved in the regulation of the cell cycle. p38 MAPK, a classical stress-responsive signaling molecule, also plays an important role in cell cycle progression [31,32]. The silkworm is an important silk-secreting insect and its silk glands undergo a unique process of endoreplication. Exploring the role of the *p38 MAPK* gene in this process may provide a molecular basis for understanding the mechanism of endoreplication. This research aims to elucidate the function of the *p38 MAPK* gene in the cell cycle and the DNA synthesis of the silkworm, highlighting its crucial role in these processes.

Previous findings in mammals suggest that *p38 MAPK* regulates G1/S and G2/M cell cycle checkpoints. In most cases, *p38 MAPK* inhibits G1 phase progression and affects the G1/S transition [33,34], while stress-activated *p38 MAPK* prevents cell transition to G2/M phase [35]. Studies in *Drosophila* also show that activated *p38 MAPK* is able to prolong the G2 phase [19]. This suggests that *p38 MAPK* functions primarily as a cell cycle repressor, consistent with our findings. Our data showed that overexpression of *Bmp38 MAPK* could significantly arrest the G1/S and G2/M phases of the cell cycle. Interestingly, interference of *Bmp38 MAPK* exhibited promotion exclusively in the G1/S phase of the cell cycle while not affecting the G2/M phase (Figure 2D, E). *p38 MAPK* normally mediates cell cycle inhibition by affecting the activity of cyclin-dependent kinases and cyclins [36,37]. We further examined G1/S checkpoint- and G2/M checkpoint-related genes, and our findings indicated that *Bmp38 MAPK* plays a regulatory role in modulating the expression levels of *BmCDK2*, *BmCyclin E*, *BmCDK1*, and *BmCyclin B* (Figure 4A–H). Meanwhile, our results showed that both overexpression and interference of *Bmp38 MAPK* could affect DNA synthesis and cell proliferation to a certain extent (Figure 2F–H). The Bmp38 MAPK protein has a highly conserved serine/threonine protein kinase catalytic domain (S-TKc), a kinase that resembles CDKs (Figure S1A). Western blotting indicated that Bmp38 MAPK has a more pronounced influence on BmCyclin B protein modification at the translation level than at the total protein level (Figure 4E–H). The inconsistent changes between transcript and protein levels of several cell cycle regulators suggest that Bmp38 MAPK may regulate cell cycle progression through both transcriptional and post-translational mechanisms. Co-IP and Y2H assays confirmed the interaction between Bmp38 MAPK and BmCyclin B and BmCDK2 (Figure 5). We hypothesize that the impact of Bmp38 MAPK inhibition on cell cycle progression may be minimal due to the intricate regulation of the cell cycle by a network of kinases and phosphatases. Further investigation is required to determine whether Bmp38 MAPK directly regulates the stability or activity of specific cyclin–CDK components through phosphorylation.

The cell cycle dynamics of silk gland cells exhibit notable variations throughout the developmental stages. During embryonic development, these cells undergo approximately 10 rounds of mitosis at stage 24, followed by a transition from mitosis to endoreplication at stage 25. Subsequently, at stage 26 and beyond, they engage in endoreplication, resulting in 17–19 rounds of genome duplication without cytokinesis. Ultimately, this process culminates in the formation of polyploid or heteroploid cells [38]. This unique cellular behavior serves as the foundation for the efficient synthesis of silk protein. Our results showed that *Bmp38 MAPK* expression was gradually downregulated in silk gland tissues of silkworm embryos at stages 24, 25 and 26, implying its involvement in silk gland DNA synthesis (Figure 1D). We further confirmed the involvement of *Bmp38 MAPK* in silk gland DNA synthesis by transient overexpression and interference of *Bmp38 MAPK* in silk gland tissues in vitro (Figure 3A–C). Previous studies had shown that the PI3K/Akt signaling pathway, the MAPK signaling pathway, the TOR signaling pathway, the Notch signaling pathway, ecdysone and insulin were involved in the regulation of DNA synthesis in the *B. mori* silk glands [39,40,41,42]. Our results supplement the regulatory network, but the specific mechanism is still unclear.

Collectively, our findings provide evidence that *Bmp38 MAPK* plays a possible role in modulating cell cycle progression and DNA synthesis in the silk glands of silkworms. It acts as a repressor of cell cycle progression, a function important for DNA synthesis in silk gland cells. Further investigations are warranted to unravel the regulatory mechanism of *Bmp38 MAPK* within the unique cell cycle environment of the silk gland.

## Data Availability

The data that support the findings of this study are available from the corresponding author upon reasonable request.

## Supplementary Materials

The following supporting information can be downloaded at: https://www.mdpi.com/article/10.3390/insects17101006/s1, Figure S1: Analysis of the sequence characteristics of Bmp38 MAPK. (A) Multiple sequence alignment of p38 MAPK across species, including a conserved S-TKc domain (red line). (B) Cell-PLoc 2.0 website predicted the subcellular localization of Bmp38 MAPK protein; Figure S2: Construction of overexpression plasmid and interference plasmid. (A) Overexpression and interference vector map. (B) Relative expression of *Bmp38 MAPK* gene after overexpression and interference in BmE-SWU1 cells. *** *p* < 0.001, paired Student’s *t*-test; Figure S3: Analysis of the interaction between Bmp38 MAPK and BmCDK1. (A) Co-localization of Bmp38 MAPK and BmCDK1 in BmE-SWU1 cells. scale bar = 10 μm. (B) Co-IP assay of Bmp38 MAPK and BmCDK1. (G) Yeast two-hybrid (Y2H) assay verified the direct interaction between Bmp38 MAPK and BmCDK1; Table S1: Primers used in this study. File S1: Full Western Blot.
