# Supplementary material for: Bombyx mori p38 MAPK Regulates Cell Cycle Progression and Silk Gland Endoreplication via Interaction with Cell Cycle Genes
Source: Insects. 2026 Sep 30;17(10):1006. doi: 10.3390/insects17101006 (PMC13704938; doi:10.3390/insects17101006)
Supplement: Supplementary file 1 [file insects-17-01006-s001.zip › insects-4575261-supplementary-Figure S1-S3, Table S1.pdf]

Supplementary Information

Table S1. Primers used in this study

Fig. S1. Analysis of the sequence characteristics of Bmp38 MAPK. (A) Multiple sequence alignment of p38 MAPK across species, including a conserved S-TKc domain (red line). (B) Cell-PLoc 2.0 website predicted the subcellular localization of Bmp38 MAPK protein.

Fig. S2. Construction of overexpression plasmid and interference plasmid. (A) Overexpression and interference vector map. (B) Relative expression of *Bmp38 MAPK* gene after overexpression and interference in BmE-SWU1 cells. ***p < 0.001, paired Student's t-test.

Fig. S3. Analysis of the interaction between Bmp38 MAPK and BmCDK1. (A) Co-localization of Bmp38 MAPK and BmCDK1 in BmE-SWU1 cells. Blue fluorescence represents the nucleus, green fluorescence represents Bmp38 MAPK, and red fluorescence represents BmCDK1; scale bar = 10 μm. (B) Co-IP assay of Bmp38 MAPK and BmCDK1. (G) Yeast two-hybrid (Y2H) assay verified the direct interaction between Bmp38 MAPK and BmCDK1.

Table S1. Primers used in this study.

Primer Name	Primer Sequence
**sw22934**	F: 5' TTCGTACTGGCTCTTCTCGT 3'
	R: 5' CAAAGTTGATAGCAATTCCCT 3'
**BmCDK2**	F: 5' CTGAAGCCCCAGAACCTACTAATA 3'
	R: 5' CGCACAGGGATACCAAAAGC 3'
**BmCyclinE**	F: 5' TCCAAGCCCTACACCTCCAA 3'
	R: 5' GCAGTCCTCATTTAGCCAGTCC 3'
**BmCDK1**	F: 5' AGGGCTCCTGAGGTCTTACT 3'
	R: 5' TGTTGGCGTTCTTAGCATTCT 3'
**BmCyclinB**	F: 5' TGTCAAAAATGTTATTCAGCC 3'
	R: 5' TTTCCGTAAAGAGTCAGTTCC 3'
**Bmp38 MAPK**	F: 5' CGCTTACGGTCAGGTTTGTT 3'
	R: 5' CGCACGATACCATCTTGTTG 3'
**Bmp38 MAPKRNAi-1**	5' CAGGTTTGTTCTGCAATAGAT 3'
**Bmp38 MAPK RNAi-2**	5' GAGACTGAAATGACAGGTTAT 3'
**Bmp38 MAPK RNAi-3**	5' ACACATGGAGCTGCCTGTAGA 3'
**Bmp38 MAPK-Flag**	F: 5' AAGGAAAAAAGCGGCCGCATGGATTACAAGGATGACGACGATAAG CCTCGTTTCCATAAAGTAGAAA 3'
	R: 5' CTAGTCTAGATTACTTATCGTCGTCATCCTTGTAATCCGATGGGTTC ACCTCGAC 3'
**BmCDK2-Myc**	F:5'CGGGATCCATGGAGCAGAAACTCATCTCTGAAGAGGATCTGGAGA ATTTTTCTACTGTAGAAA 3'
	R:5'GCTCTAGATTACAGATCCTCTTCAGAGATGAGTTTCTGCTCAGGAT GTAGAGGAGGCGGCACC 3'
**BmCyclinB-HA**	F:5'GGGGTACCATGTACCCATACGACGTCCCAGACTACGCTGAAATTCA ACTAAGGCGGCAT 3'
	R:5'GGACTAGTCTAAGCGTAGTCTGGGACGTCGTATGGGTAAGGGCTTG GGCTTGATATCT 3'
**BmCyclinE-Myc**	F:5'GGGGTACCATGGAGCAGAAACTCATCTCTGAAGAGGATCTGGCTG AATCTGGGTCTCCC 3'
	R:5'GGACTAGTTCACAGATCCTCTTCAGAGATGAGTTTCTGCTCGTCAG CATGTATCCTTAG 3'
**BmCDK1-HA**	F:5'ATTTGCGGCCGCATGTACCCATACGATGTTCCAGATTACGCTGATGA TTTCTTAAAGAT 3'
	R:5'GCTCTAGATTAAGCGTAATCTGGAACATCGTATGGGTATACACTTTG AACAGAATCCGT 3'
**Bmp38 MAPK-BD**	F:5' CGGAATTCATGCCTCGTTTCCATAAAGTAGAAA 3'
	R:5' CGGGATCC TTACGATGGGTTCACCTCGAC 3'
**BmCDK2-AD**	F:5' CCATCGATATGGAGAATTTTTCTACTGTAGAAA 3'
	R:5' CGGGATCCTTAAGGATGTAGAGGAGGCGGCACC 3'
**BmCyclinB-AD**	F:5' CCATCGATATGGAAATTCAACTAAGGCGGCATA 3'
	R:5' CGGGATCC CTAAGGGCTTGGGCTTGATATCT 3'

**BmCyclinE-AD**	F:5' CCATCGATATGGCTGAATCTGGGTCTCCCTCTG 3'
	R:5' CGGGATCC TCAGTCAGCATGTATCCTTAGTTCG 3'
**BmCDK1-AD**	F:5' CCATCGATATGGATGATTTCTTAAAGATAGAAA 3'
	R:5' CCGCTCGAGCTATACACTTTGAACAGAATCCGTG 3'

A

B

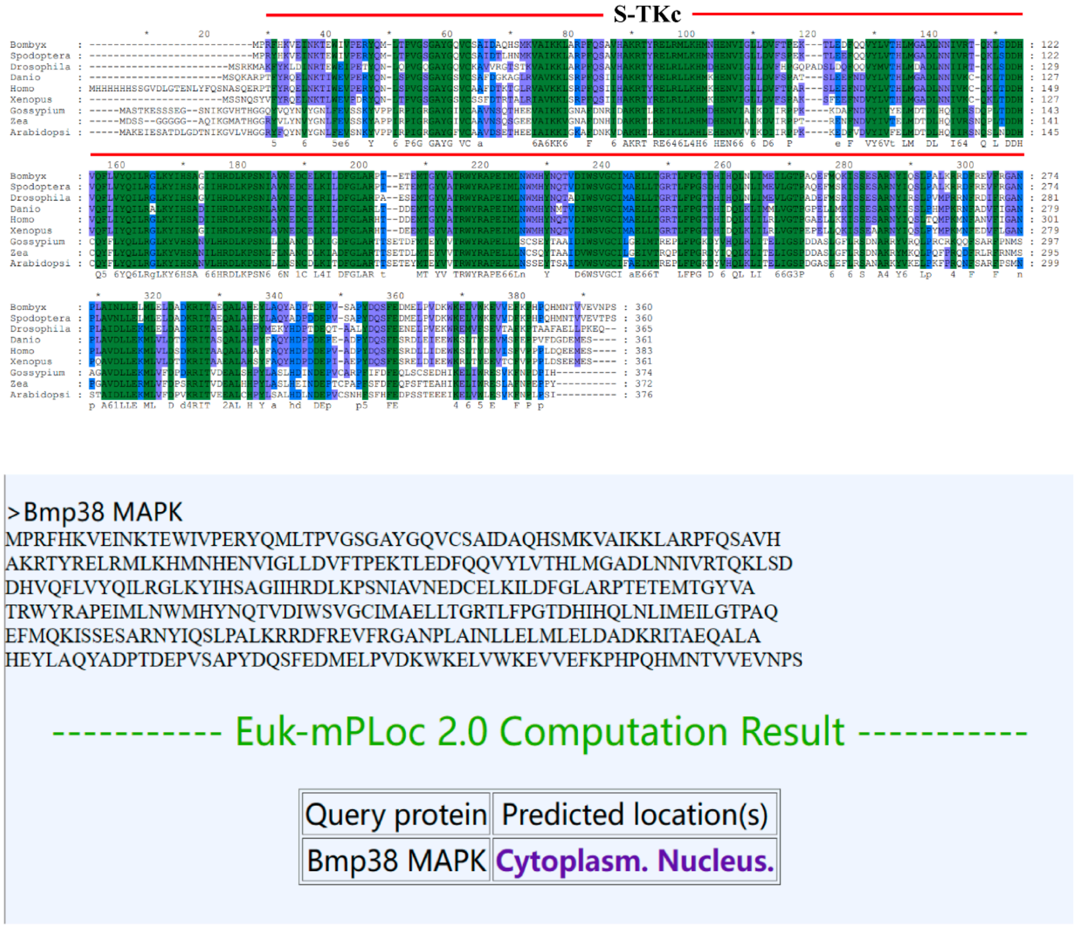

Fig. S1. Analysis of the sequence characteristics of Bmp38 MAPK.

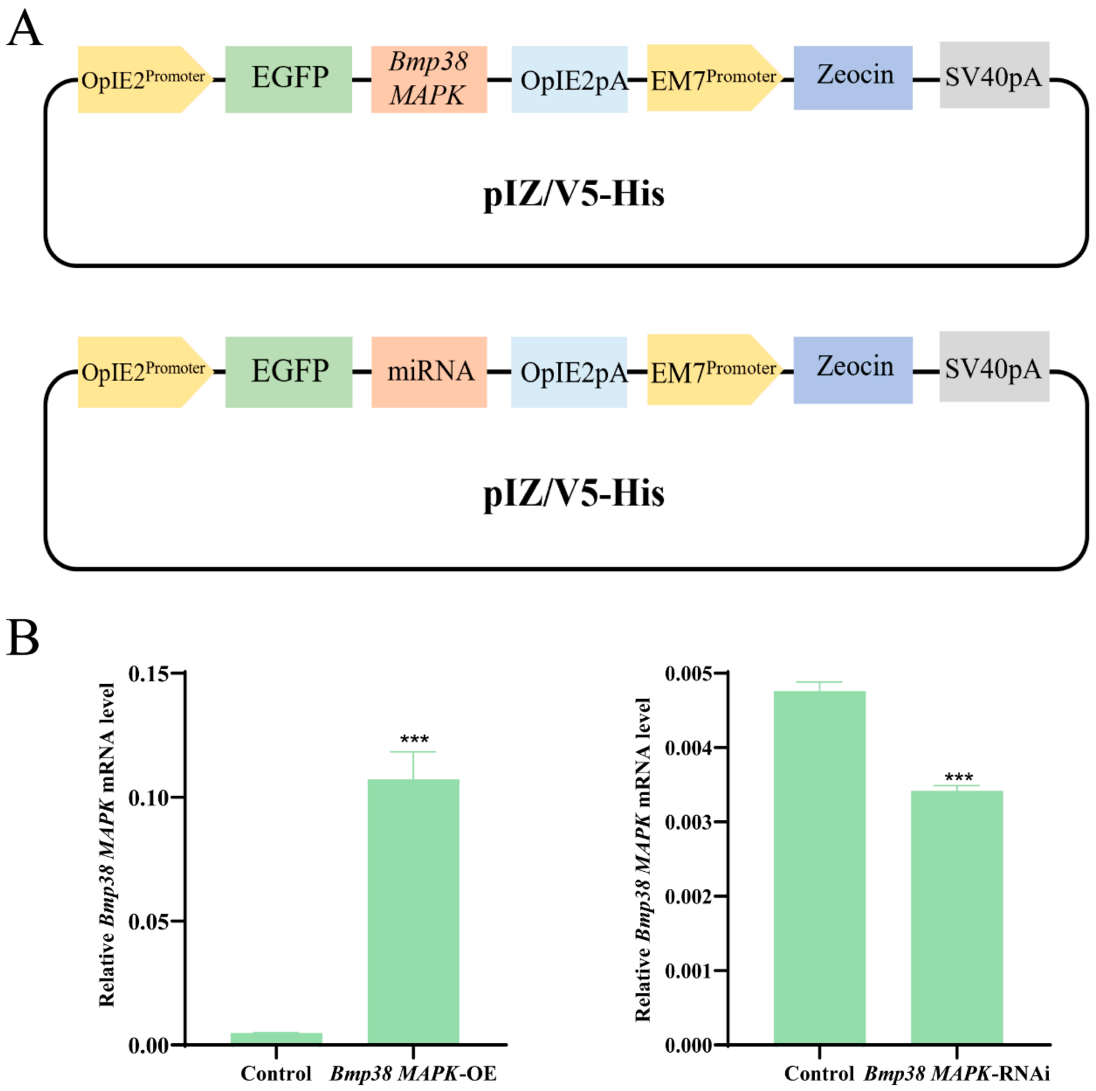

Fig. S2. Construction of overexpression plasmid and interference plasmid.

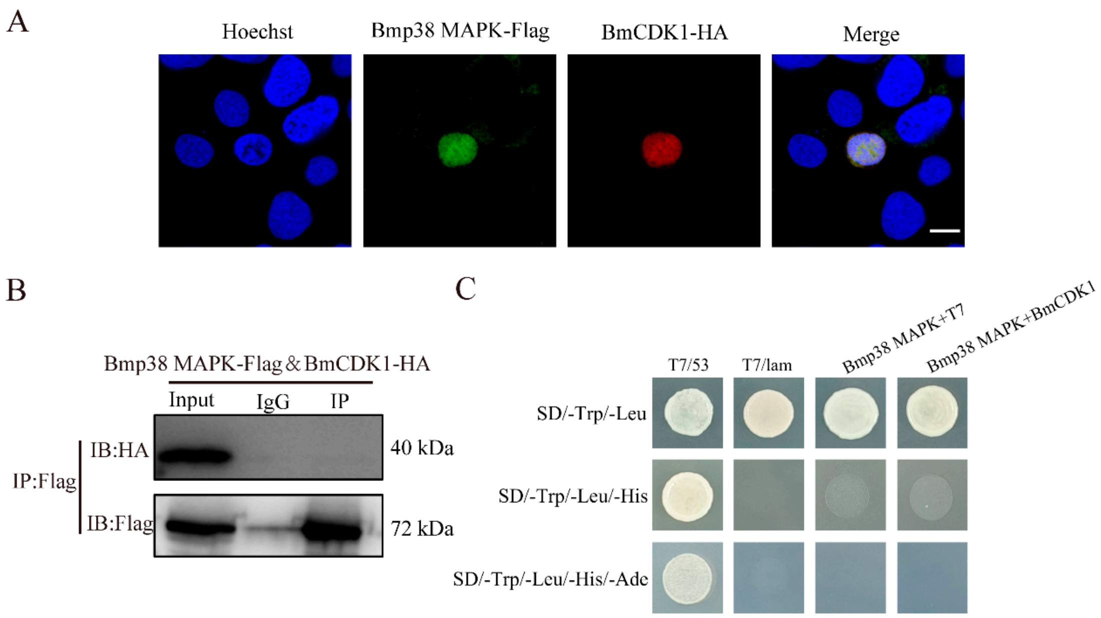

Fig. S3. Analysis of the interaction between Bmp38 MAPK and BmCDK1.
