# Supplementary material for: Bombyx mori p38 MAPK Regulates Cell Cycle Progression and Silk Gland Endoreplication via Interaction with Cell Cycle Genes
Source: Insects. 2026 Sep 30;17(10):1006. doi: 10.3390/insects17101006 (PMC13704938; doi:10.3390/insects17101006)

BmCDK1 for Figure 4E

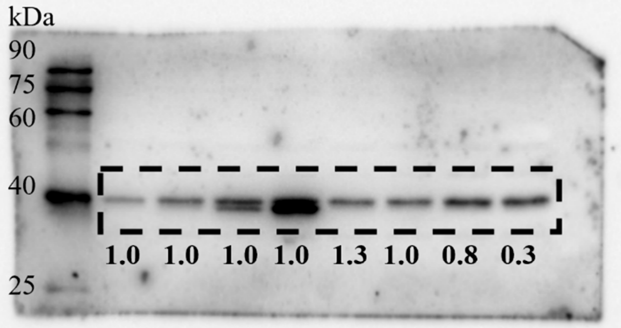

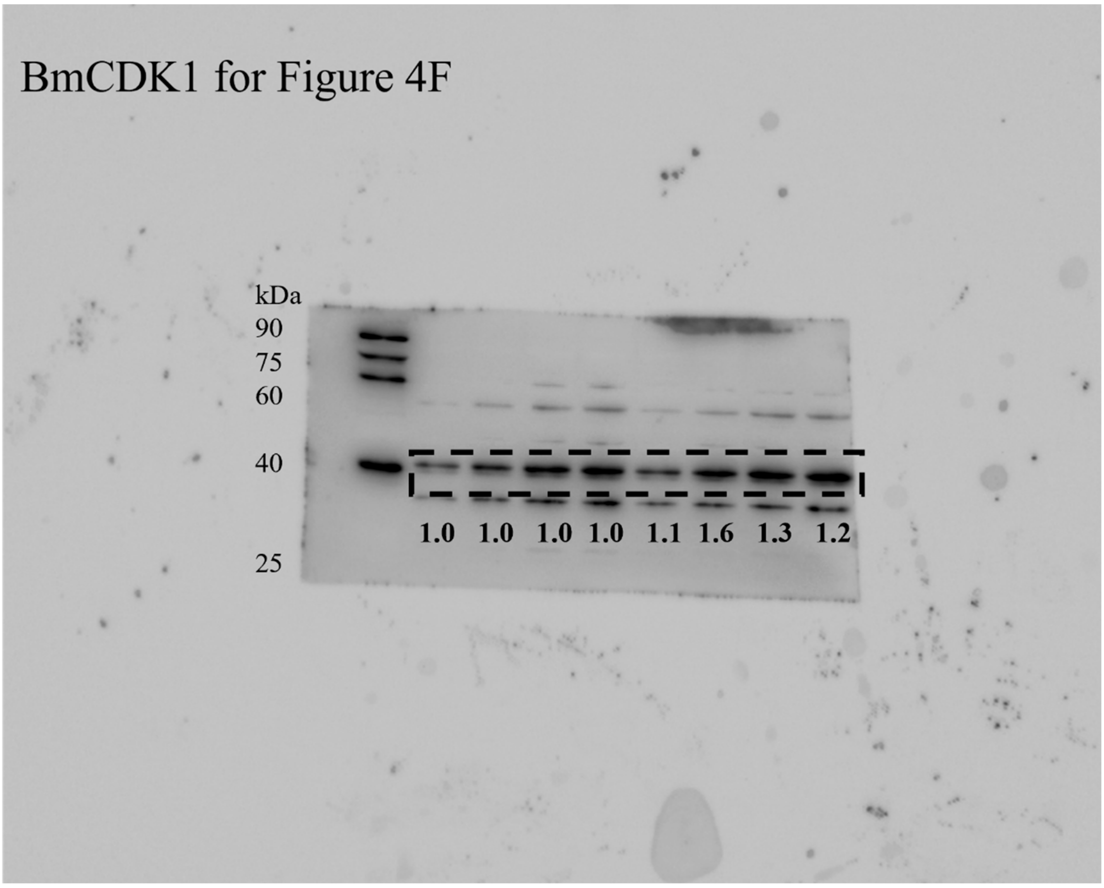
BmCDK1 for Figure 4F
kDa
90
75
60
40
25
1.0
1.0
1.0
1.0
1.1
1.6
1.3
1.2

BmCyclin E for Figure 4E

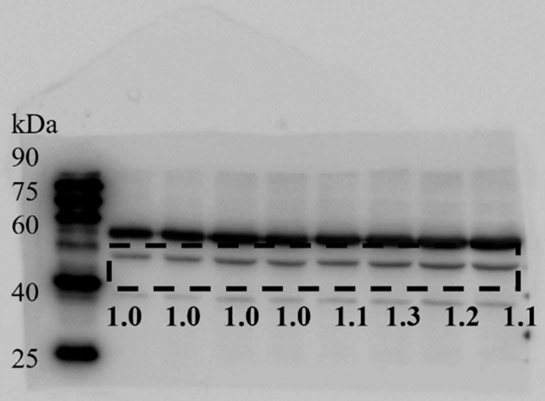

BmCyclin E for Figure 4F

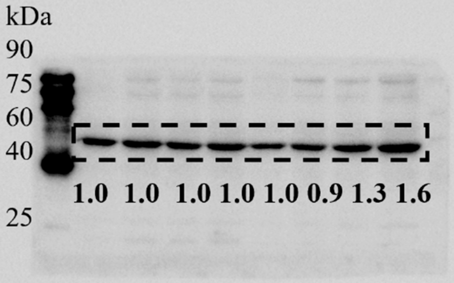

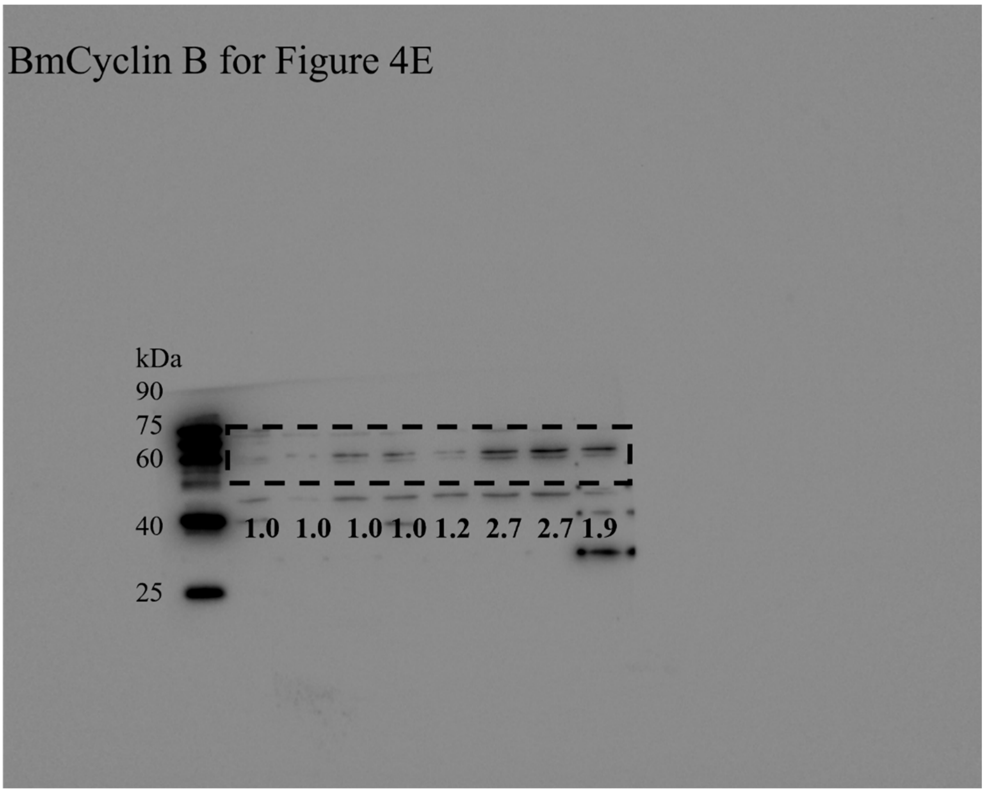
BmCyclin B for Figure 4E
kDa
90
75
60
40
25
1.0 1.0 1.0 1.0 1.2 2.7 2.7 1.9

BmCyclin B for Figure 4F

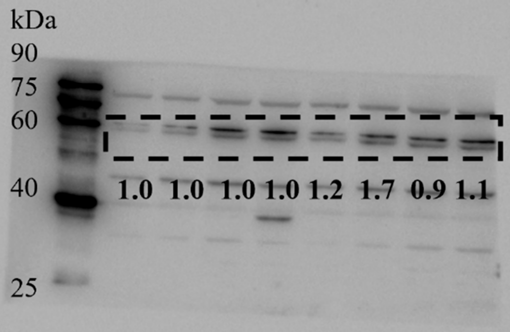

α-Tubulin for Figure 4E

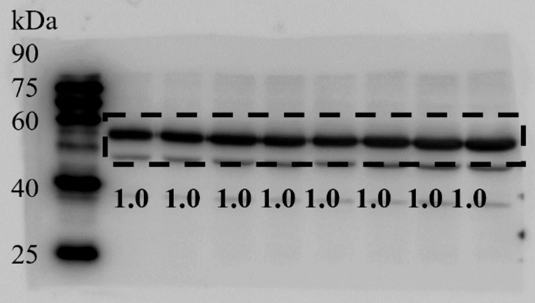

α-Tubulin for Figure 4F

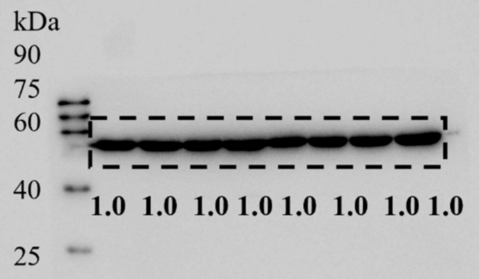

Myc for Figure 5D

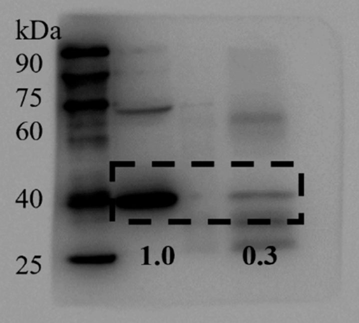

Flag for Figure 5D

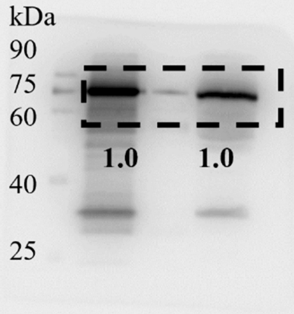

Myc for Figure 5E

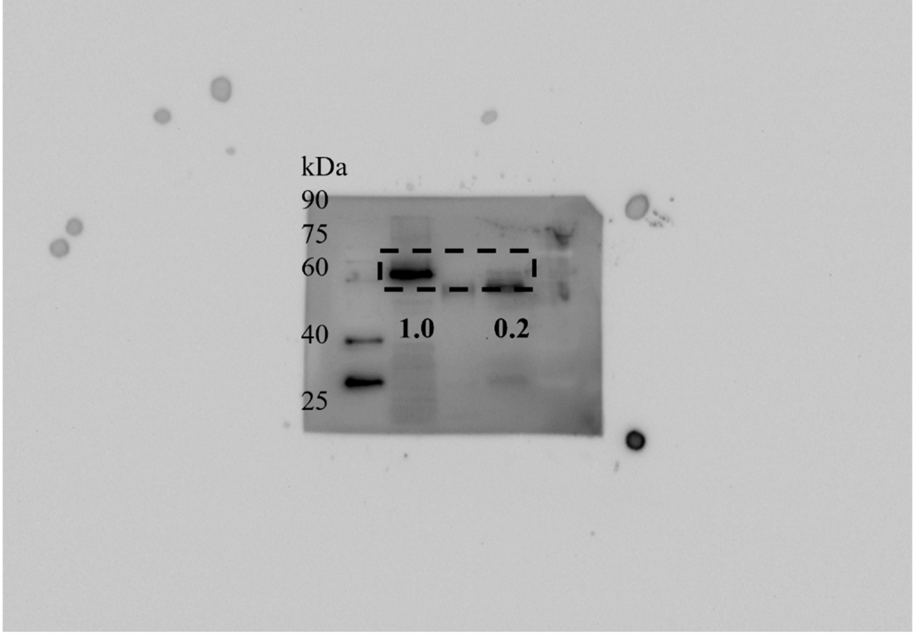

Flag for Figure 5E

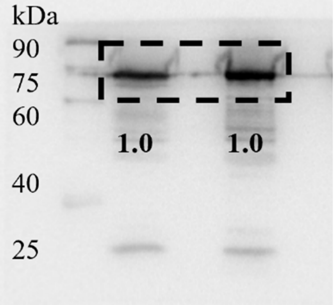

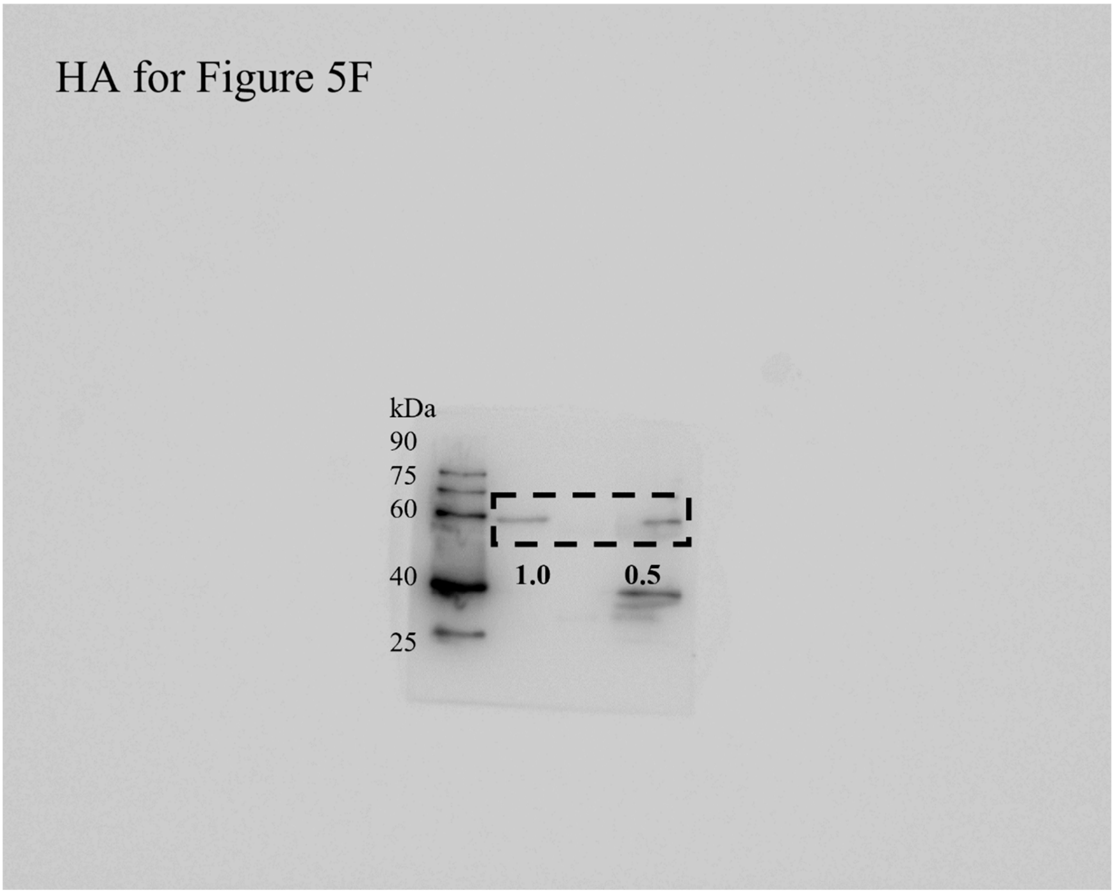
HA for Figure 5F
kDa
90
75
60
40
25
1.0
0.5

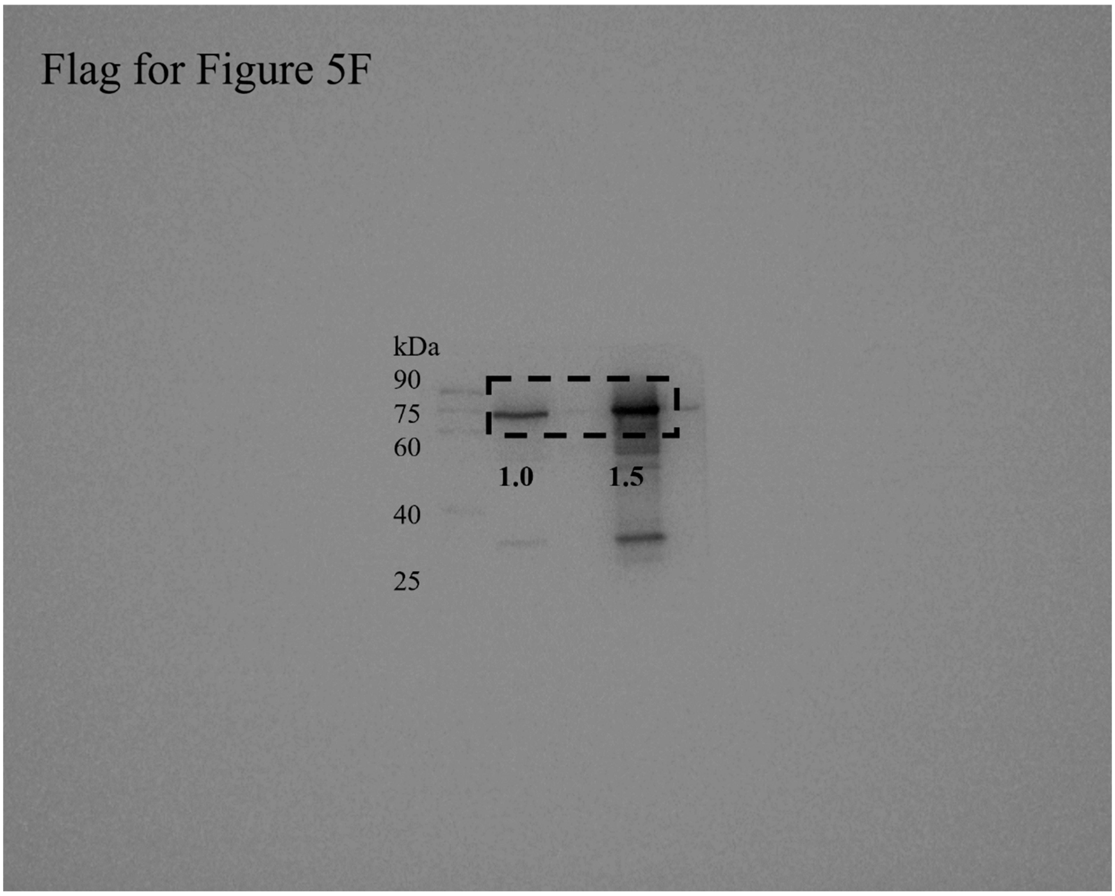
Flag for Figure 5F
kDa
90
75
60
40
25
1.0
1.5

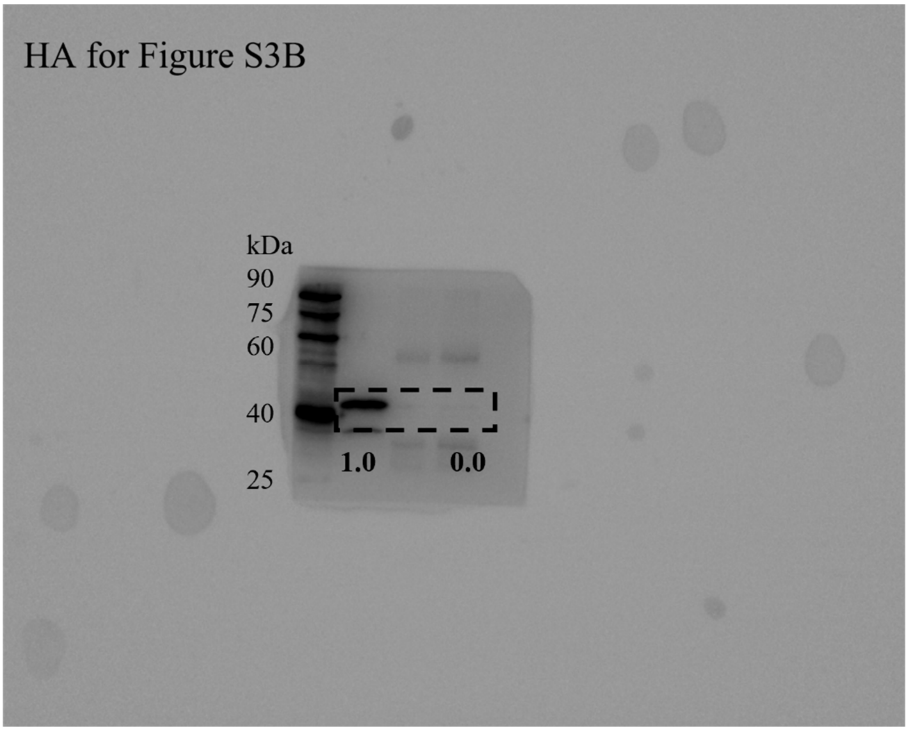
HA for Figure S3B
kDa
90
75
60
40
25
1.0
0.0

Flag for Figure S3B

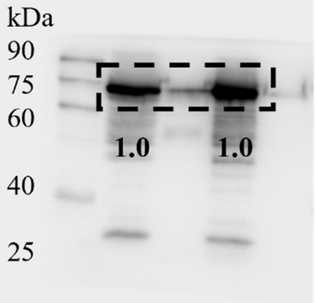

Supplement: Supplementary file 1 [file insects-17-01006-s001.zip › insects-4575261-supplementary-file S1-Full Western Blot.pdf]
